# Early Events in Japanese Encephalitis Virus Infection: Viral Entry

**DOI:** 10.3390/pathogens7030068

**Published:** 2018-08-13

**Authors:** Sang-Im Yun, Young-Min Lee

**Affiliations:** Department of Animal, Dairy, and Veterinary Sciences, College of Agriculture and Applied Sciences, Utah State University, Logan, UT 84322, USA; sangim.yun@usu.edu

**Keywords:** Japanese encephalitis virus, flavivirus, viral replication, viral entry, attachment, binding, endocytosis, internalization, membrane fusion, virus-host interaction

## Abstract

Japanese encephalitis virus (JEV), a mosquito-borne zoonotic flavivirus, is an enveloped positive-strand RNA virus that can cause a spectrum of clinical manifestations, ranging from mild febrile illness to severe neuroinvasive disease. Today, several killed and live vaccines are available in different parts of the globe for use in humans to prevent JEV-induced diseases, yet no antivirals are available to treat JEV-associated diseases. Despite the progress made in vaccine research and development, JEV is still a major public health problem in southern, eastern, and southeastern Asia, as well as northern Oceania, with the potential to become an emerging global pathogen. In viral replication, the entry of JEV into the cell is the first step in a cascade of complex interactions between the virus and target cells that is required for the initiation, dissemination, and maintenance of infection. Because this step determines cell/tissue tropism and pathogenesis, it is a promising target for antiviral therapy. JEV entry is mediated by the viral glycoprotein E, which binds virions to the cell surface (attachment), delivers them to endosomes (endocytosis), and catalyzes the fusion between the viral and endosomal membranes (membrane fusion), followed by the release of the viral genome into the cytoplasm (uncoating). In this multistep process, a collection of host factors are involved. In this review, we summarize the current knowledge on the viral and cellular components involved in JEV entry into host cells, with an emphasis on the initial virus-host cell interactions on the cell surface.

## 1. Introduction: JEV Is a Mosquito-Borne Neurotropic Flavivirus

Japanese encephalitis virus (JEV) is a member of the genus *Flavivirus*, family *Flaviviridae* [1,2]. Most flaviviruses replicate in both hematophagous arthropod vectors (i.e., mosquitoes and ticks) and vertebrate animal hosts (e.g., mammals and birds) [3,4,5,6,7], but some infect only arthropods (e.g., mosquitoes and sand flies) or almost exclusively vertebrates (e.g., bats and rodents) [8,9,10,11,12]. Based on the host range and choice of vector species, flaviviruses can be divided into four groups [10,11,12]: mosquito-borne, tick-borne, arthropod-restricted, and vertebrate-restricted viruses, of which the last group is commonly referred to as no known vector viruses. Many of the mosquito- and tick-borne flaviviruses are the major emerging and re-emerging pathogens that present a global challenge to human and animal medicine [13,14,15,16]. Of the mosquito-borne flaviviruses, JEV is the prototype member of the Japanese encephalitis (JE) serogroup [17] that also includes the West Nile virus (WNV), Murray Valley encephalitis virus (MVEV), St. Louis encephalitis virus (SLEV), and four other lesser known flaviviruses, namely the Usutu virus, Koutango virus, Yaounde virus, and Cacipacore virus [18,19]. Although antigenically distinct, JEV is genetically close to several medically important mosquito-borne flaviviruses, such as the Zika virus (ZIKV), dengue virus (DENV), and yellow fever virus (YFV) [19,20,21], as well as the tick-borne encephalitis virus (TBEV) [22].

JEV is the etiological agent of JE, a serious neurological disease characterized by extensive inflammation in the central nervous system [23,24]. JE is the most common form of viral encephalitis occurring in the Asia-Pacific region, particularly in southern, eastern, and southeastern Asia, as well as northern Oceania [25,26,27,28,29]. Initially seen in Japan, outbreaks of “summer encephalitis”, presumably caused by JEV infection, were described as early as 1871, but it was not until 1924 that the first cases of JE were diagnosed [30]. Since then, JEV has become prevalent in much of Asia, with a fatality rate of up to ~30% [31] despite multiple JE vaccines having been made commercially available in this region [32]: Its geographic boundaries have continued to expand southward into Papua New Guinea [33,34] and Australia [35,36,37,38,39,40], eastward through the Pacific Islands [41], and westward into Pakistan [42] and China (Tibet) [43,44]. Surprisingly, in Italy, JEV RNA was detected by RT-PCR assays in dead birds during 1997–2000 and field-collected mosquitoes in 2010 [45,46], raising concern regarding the long-distance spread of the virus from the Asia-Pacific region and its potential autochthonous transmission in Europe [47]. Likewise, the emergence of JEV in the Western Hemisphere is also conceivable [48].

## 2. JEV Is a Zoonotic Pathogen Capable of Infecting a Wide Range of Animal Species

JEV is transmitted among multiple vertebrate hosts primarily through the bite of an infected mosquito. In most Asian countries, *Culex tritaeniorhynchus* is known as the primary mosquito vector for JEV transmission [49,50,51,52,53,54]; in Australia, on the other hand, *Cx*. *annulirostris* is identified as the main vector involved in the introduction and spread of JEV [36,37,38,55]. Also, JEV has been isolated or detected, albeit at various frequencies, in other wild-caught *Culex* mosquitoes (e.g., *Cx. annulus*, *Cx. bitaeniorhynchus*, *Cx. fuscocephala*, *Cx. gelidus*, *Cx. orientalis*, *Cx. pipiens*, *Cx. pseudovishnui*, *Cx. quinquefasciatus*, and *Cx. vishnui*), suggesting that they may play a role in local JEV transmission [45,50,52,53,55,56,57,58,59,60,61,62,63,64]. Similarly, recent experimental studies on the vector competence of European mosquitoes have shown that *Cx. pipiens* and three *Aedes* species (*Ae. albopictus*, *Ae. detritus*, and *Ae. japonicus*) are susceptible to JEV infection in a laboratory setting [65,66,67]. Moreover, JEV infection has been detected in field-collected or experimentally inoculated non-*Culex* mosquitoes, such as *Ae. albopictus*, *Ae. vexans*, *Armigeres subalbatus*, and *Mansonia uniformis*, and three *Anopheles* species (*An. minimus*, *An. sinensis*, and *An. tessellatus*), raising the question of whether they can act as potential vectors under certain environmental conditions [50,68,69]. Furthermore, JEV can be passed directly from an infected female *Culex* or non-*Culex* mosquito to her eggs, suggesting the transovarial transmission as a mechanism by which the virus overwinters in the environment [70,71,72,73]. In addition, in terms of non-vector-borne transmission, a recent report has indicated that JEV can be transmitted through the transfusion of contaminated blood products [74].

The natural cycle of JEV involves numerous vertebrate hosts. In Asia, domestic pigs and water birds have been recognized as the two most important JEV-amplifying hosts, since they are generally asymptomatic following infection, but develop high-titer viremias sufficient to transmit the virus to engorging mosquitoes [75,76,77,78,79,80,81,82]. In sows, it is noteworthy that JEV infection during pregnancy often causes abortions and stillbirths [83,84,85,86]. Bats, along with migratory birds, may play a role in the overwintering and dispersal of JEV, as suggested by detection of the virus and its IgG antibody [87,88,89,90,91,92]. On the other hand, horses, like humans, are considered to be incidental hosts that sometimes develop fatal encephalitis following JEV infection, but are not believed to be a significant source of the virus for mosquitoes, although they may occasionally develop viremia that allows mosquito infection, because of their small population size and long generation time [93,94,95,96,97,98,99,100,101]. In cows, JEV rarely causes neurological disorders [102,103,104,105,106], and little or no viremia is typically detected [107]. Serological surveys and experimental infection studies have suggested that JEV can subclinically infect other vertebrate animals, such as dogs, goats, sheep, buffaloes, boars, raccoons, raccoon dogs, ducks, and chickens [108,109,110,111,112,113,114], underlining the need to investigate their potential roles in JEV ecology [115]. Notably, ducklings and chicks under two weeks of age have been shown to develop considerably high viremias following JEV infection, but the development of viremia is inversely correlated with the age of the animals at infection [116]. Interestingly, JEV-infected pigs are demonstrated to shed the virus in oronasal secretions [117] and transmit it to co-housed naive pigs in the absence of mosquitoes, suggesting a mode of viral transmission during mosquito-free seasons [118]. Further studies are needed to understand the dynamic interactions between the virus, mosquito vectors, and vertebrate hosts under certain geo-environmental and eco-agricultural conditions [119,120,121].

## 3. JEV Is a Small Enveloped Positive-Strand RNA Virus

### 3.1. Genome Structure and Gene Expression

**Figure 1:**
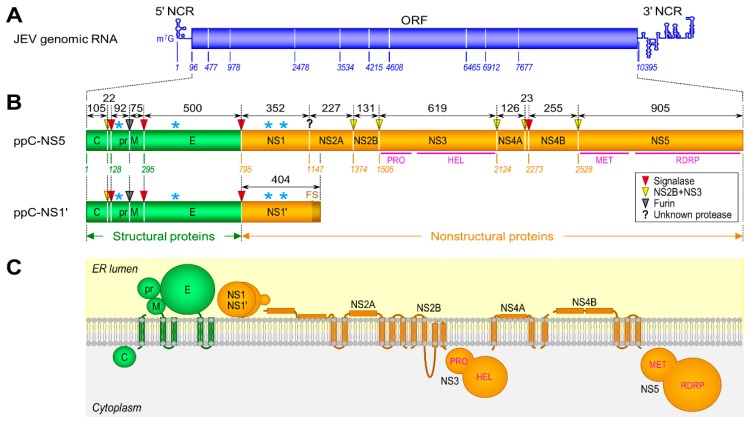
Genome organization and gene expression of Japanese encephalitis virus (JEV). (**A**) Genome organization. The genetically well-characterized JEV strain, CNU/LP2, contains a single-stranded positive-sense RNA genome of 10,968 nucleotides in length, consisting of a methylated cap at the 5′ end, followed by a 95-nucleotide 5′ non-coding region (5′NCR), a 10,299-nucleotide open reading frame (ORF), and a 574-nucleotide 3′NCR [164,165]. (**B**) Gene expression. The single ORF encoded in the viral genome produces two precursor polyproteins, a full-length 3432-amino acid polyprotein (ppC-NS5) and its C-terminally truncated 1198-amino acid polyprotein (ppC-NS1′), the latter of which is expressed by a −1 ribosomal frameshift (FS) event that occurs between the codons 8 and 9 of NS2A, adding 52 extra amino acids to the C-terminus of NS1 (designated NS1′). Each of the two polyproteins is cleaved by host- and virus-encoded proteases to yield three structural (green) and at least seven nonstructural (orange) proteins. The flavivirus-conserved cleavage sites and the length of cleavage products are indicated. NS3 consists of an N-terminal serine protease (PRO) domain and a C-terminal RNA helicase (HEL) domain, and NS5 consists of an N-terminal methyltransferase (MET) domain and a C-terminal RNA-dependent RNA polymerase (RDRP) domain. Asterisks indicate four N-linked glycosylation sites (NXT/S) found in the pr portion of prM (Asn^15^), E (Asn^154^), and NS1/NS1′ (Asn^130^ and Asn^207^). (**C**) Polyprotein membrane topology. The membrane orientation of the 10 major JEV proteins is predicted on the endoplasmic reticulum (ER) membrane, based on previous work with other flaviviruses [142,166,167,168,169].

JEV is an enveloped RNA virus with a linear, single-stranded, and positive-sense RNA genome of ~11 kb in length (Figure 1A). The genomic RNA has a methylated cap structure at its 5′ end, but lacks a poly (A) tail at the 3′ end [122,123,124,125,126]. It has one long open reading frame (ORF) encoded between the two short, but highly structured, 5′ and 3′ non-coding regions (NCRs) that form a long-range intramolecular RNA-RNA interaction to regulate viral translation and RNA replication [127,128,129,130,131]. In addition to the viral genomic RNA, a group of short non-coding subgenomic RNAs (~0.2–0.5 kb) is also accumulated to high levels in a diverse range of mammalian and insect cells infected with JEV and other flaviviruses as a result of incomplete degradation of the genomic RNA caused by the stalling of the cellular 5′→3′ exoribonuclease Xrn1 just upstream of a higher-order structure in the 3′NCR [132,133,134,135,136]. The generation of this subgenomic RNA may cause the suppression of Xrn1 and the dysregulation of cellular mRNA stability [137], thereby disrupting the host’s innate immune responses and contributing to viral replication and pathogenesis [138,139,140].

The ORF in the JEV genomic RNA encodes a polyprotein precursor of ~3432 amino acids, which is cleaved into at least 10 distinct products [141,142], i.e., three structural (capsid, C; premembrane, prM; and envelope, E) and seven nonstructural (NS1, NS2A, NS2B, NS3, NS4A, NS4B, and NS5) proteins (Figure 1B,C). In flaviviruses, the site-specific proteolysis of the polyprotein is catalyzed co- and post-translationally by a set of four different proteases: (i) the host signal peptidase responsible for cleaving at the C-prM, prM-E, E-NS1, and NS4A-NS4B junctions within the lumen of the endoplasmic reticulum (ER) [143,144,145,146,147,148]; (ii) the two-component viral protease NS3 + NS2B [149] required for cleaving at the NS2A-NS2B, NS2B-NS3, NS3-NS4A, and NS4B-NS5 junctions, as well as at internal sites within the C and NS4A proteins on the cytoplasmic face of the ER membrane [143,144,150,151,152,153,154,155]; (iii) the host furin or furin-like protease mediating the final cleavage of prM to M in the *trans*-Golgi network [156]; and (iv) an unknown host protease capable of cleaving at the NS1-NS2A junction [157,158,159]. In addition to the aforementioned 10 proteins, an NS1 isoform (NS1′) is also produced during infection with JEV and other JE serogroup members as a result of −1 translational frameshifting occurring at codons 8–9 of NS2A [160,161,162,163].

### 3.2. Viral Replication Cycle

**Figure 2:**
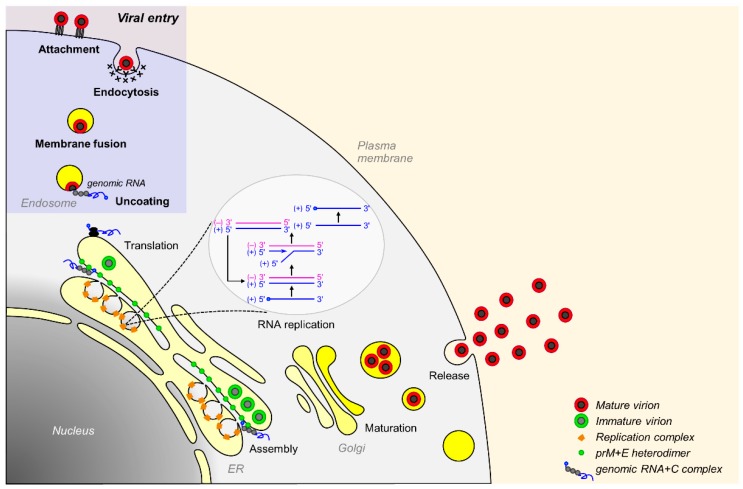
Replication cycle of JEV. A viral particle binds initially to a target cell through one or more attachment factors on the cell surface (Attachment). Subsequently, the virion interacts with an entry factor(s), which triggers receptor-mediated clathrin-dependent or clathrin-independent endocytosis of the bound virion (Endocytosis). Following internalization, the virion travels through the endosomal maturation pathway until endosomal acidification triggers the low pH-induced activation of the viral E glycoprotein, enabling the fusion between viral and endosomal membranes (Membrane fusion). Upon release of the viral genomic RNA into the cytoplasm (Uncoating), it is translated into two overlapping polyproteins as the result of a −1 ribosomal frameshift event in association with the ER (Translation). The polyproteins are co- and post-translationally cleaved to generate the mature viral proteins that are essential for RNA replication and virion assembly. The genomic RNA is replicated in the replication complex within a structurally rearranged ER-derived membrane vesicles (RNA replication). Viral assembly proceeds with the concomitant interaction of viral RNA with the three structural proteins (C, prM, and E), promoting the budding of immature particles into the lumen of the ER (Assembly). The immature virions, containing heterodimers of prM and E, are then transported through the *trans*-Golgi network, where prM is cleaved to form mature virions containing homodimers of M and E (Maturation). Finally, both partially and completely mature virions are released into the extracellular milieu (Release).

JEV is a flavivirus containing an inner nucleocapsid [170], a disordered structure made of the genomic RNA and helix-rich C proteins [171,172,173]. The nucleocapsid is enclosed by a lipid bilayer, which is in turn encased in a well-organized outer protein shell composed of the membrane-anchored prM/M and E proteins [174,175,176,177,178,179]. Basically, JEV shares a common strategy for viral replication with other flaviviruses (Figure 2). Viral entry is a dynamic process, defined by a series of interactions between the virus and the host cell that starts with nonspecific binding of the viral glycoprotein E to one or more cellular attachment factors on the cell surface [180,181,182,183,184,185]. This attachment step serves to concentrate the virions at the cell surface to facilitate the specific interaction of the viral E glycoprotein with a cellular entry factor(s) [186,187,188], directing the classical clathrin-dependent endocytosis [189,190,191,192,193,194,195,196,197,198,199,200] or non-classical clathrin-independent endocytosis pathways [201,202,203,204,205,206], presumably in a cell type-restricted fashion. Once inside the endosome, the viral E glycoprotein undergoes low pH-induced conformational changes [207,208,209,210,211,212,213], triggering the fusion of viral and host endosomal membranes [214,215,216,217,218,219,220,221]. Following membrane fusion, the genomic RNA is released into the cytoplasm, where it is translated into two precursor polyproteins (with or without a ribosomal frameshifting at the beginning of NS2A-coding region) that are cleaved to yield three structural (C, prM, and E) and at least seven nonstructural (NS1 to NS5) proteins, along with NS1′ [141,142].

After translation, all seven nonstructural proteins, together with the poorly understood host factors [222], are involved directly or indirectly in the genomic RNA replication that occurs in the virus-induced ER-derived membraneous organelle [223,224,225,226,227] housing the replication complexes [123,228]. Viral RNA replication is catalyzed by NS3 and NS5 [229], the two largest and most conserved nonstructural proteins that coordinate their multiple enzymatic activities in negative-strand RNA synthesis, positive-strand RNA synthesis, RNA capping, and cap methylation [123,149,230]. During or shortly after RNA replication, a complex of the newly synthesized genomic RNA and C proteins is enveloped by two viral glycoproteins (prM and E [231]) on the ER membrane to produce the immature virion (~60 nm diameter) covered with 60 protruding spikes, each composed of three parallel prM:E heterodimers [232,233,234,235]. The immature virions are believed to pass through the constitutive secretory pathway to the extracellular space. During this exocytosis, viral maturation occurs in the *trans*-Golgi network through the furin-mediated cleavage of the prM protein to M [156,236,237,238], accompanied by a significant structural rearrangement of the M and E proteins, to generate the mature virion (~50 nm diameter), which is covered by 30 flat densely packed rafts, each composed of three parallel E:M:M:E heterotetramers [232,239,240]. In addition to the M-containing completely mature virions, prM-containing partially mature, but still infectious, virions are also shown to be produced [241,242,243,244], although viral infectivity is likely compromised [245]. Overall, viral replication takes place entirely in the cytoplasm; however, two viral proteins, C [246,247,248,249,250] and NS5 [251,252,253,254,255,256,257], are not only detected in the cytoplasm, but are also found in the nucleus [258]. The precise role of their nuclear localization in viral replication and pathogenesis requires further investigation.

## 4. Viral Entry Is the First Step in the Infection Process

Viral entry is the first step in an orchestrated process of virus-host interactions that is not only required for the initiation, dissemination, and maintenance of productive infection [259,260], but also represents a critical determinant of cell/tissue tropism and pathogenesis [261]. JEV entry is thus a promising target for antiviral therapy and offers multiple points for intervention [262]: attachment, endocytosis, membrane fusion, and uncoating (see a recent review article for a detailed description of small-molecule inhibitors targeting flavivirus entry [263]). Identifying the viral and host factors involved in JEV entry is a prerequisite to elucidating the molecular mechanisms of viral entry and developing novel therapeutic and preventive antivirals. In recent years, tremendous progress has been made in understanding the viral components required for the various steps of JEV entry, but little is known about the cellular components involved in this important process.

### 4.1. Virus Structure

**Figure 3:**
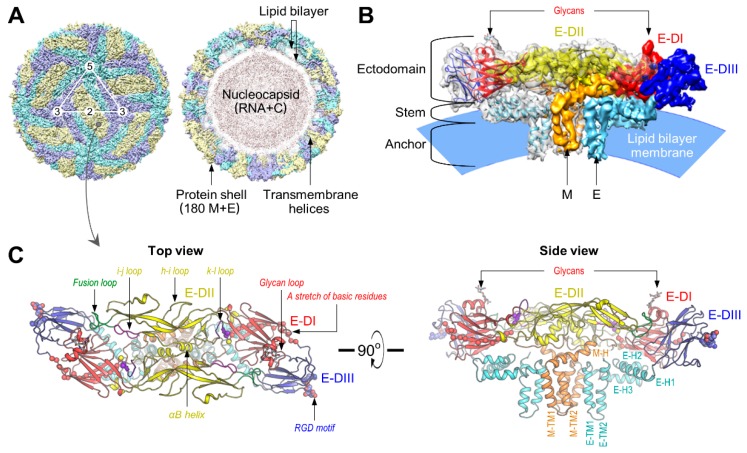
The cryo-electron microscopy (EM) structure of JEV at 4.3 Å resolution. (**A**) The surface (left) and cross-section (right) of the cryo-EM map of mature JEV (strain P3). An icosahedral asymmetric unit is indicated by a white triangle, with the 2-, 3-, and 5-fold symmetry axes labeled. E:M heterodimers of the same color are equivalent by icosahedral symmetry. (**B**) Side view of the E:M heterodimer fitted into the electron density map. Indicated are the ectodomain, stem, and anchor regions of the E protein, along with the M protein buried underneath it. (**C**) Ribbon diagram of the E:M:M:E heterotetramer. Color-coded are the structurally distinct domains/regions of E and M proteins: E-DI (domain I), E-DII, E-DIII, E-H1 (helix 1), E-H2, E-H3, E-TM1 (transmembrane 1), E-TM2, M-H, M-TM1, and M-TM2. Also highlighted are the functionally important structural components in the E ectodomain: glycan loop and a stretch of basic residues in E-DI; fusion loop, h-i loop, i-j loop, k-l loop, and αB helx in E-DII; and Arg-Gly-Asp (RGD) motif in E-DIII. In all cases, one molecule is used for labeling. The high-resolution images of this figure were kindly provided by Dr. Xiangxi Wang [176].

Using cryo-electron microscopy (EM) and image reconstruction techniques, Wang and coworkers have determined the 4.3-Å three-dimensional structure of JEV [176]. On the surface of the mature JEV, 180 copies of each of the M and E proteins are organized into 30 flat, densely packed rafts. Each of these rafts is composed of three parallel E:M:M:E heterotetramers, with the E proteins forming the smooth outer protein shell and the M proteins being buried underneath it (Figure 3A), as seen in the cryo-EM structures initially of DENV [174,179] and WNV [175], and lately ZIKV [177,178]. The JEV E monomer, like that of other flavivirus E proteins, consists of three topologically distinct segments (Figure 3B,C): (i) a banana-shaped ectodomain, which mediates receptor binding and membrane fusion; (ii) a “stem” region, which includes three perimembrane helices lying nearly horizontal on the viral membrane underneath the ectodomain; and (iii) an “anchor” region, which contains two antiparallel membrane-embedded helices [240,264,265,266]. Notably, the E ectodomain adopts a three-domain architecture, with domain I (E-DI) lying at the interface between domains II (E-DII) and III (E-DIII) (Figure 3C): (a) E-DI has the glycan loop carrying an N-linked carbohydrate chain attached to Asn^154^ and a string of six closely dispersed basic residues (Lys^279^ to Lys^297^) mapped in the last strand I_0_ of E-DI and the linker between E-DI and DIII; (b) E-DII contains the fusion loop at its tip and several potentially functionally important loops (e.g., h-i, i-j, and k-l loops) on its side; and (c) E-DIII has the Arg-Gly-Asp (RGD) motif and is implicated in receptor binding and antibody neutralization [267,268,269,270,271,272,273,274,275,276,277,278,279,280,281,282,283,284]. In contrast, the JEV M monomer contains a flexible N-terminal loop, followed by an amphipathic helix lying on the membrane and two antiparallel helices embedded in the membrane (Figure 3C). The N-terminal loop of M participates in electrostatic and hydrophobic interactions with E-DI and E-DII, and the amphipathic helix of M is involved in hydrogen-bond interactions with E-DII (centered at Gln^264^ near helix αB) and the N-terminus of a neighboring M. The E-DI, E-DIII, and the helical stem region of E are held together by charge interactions.

### 4.2. Attachment

#### 4.2.1. Viral Components

**Figure 4:**
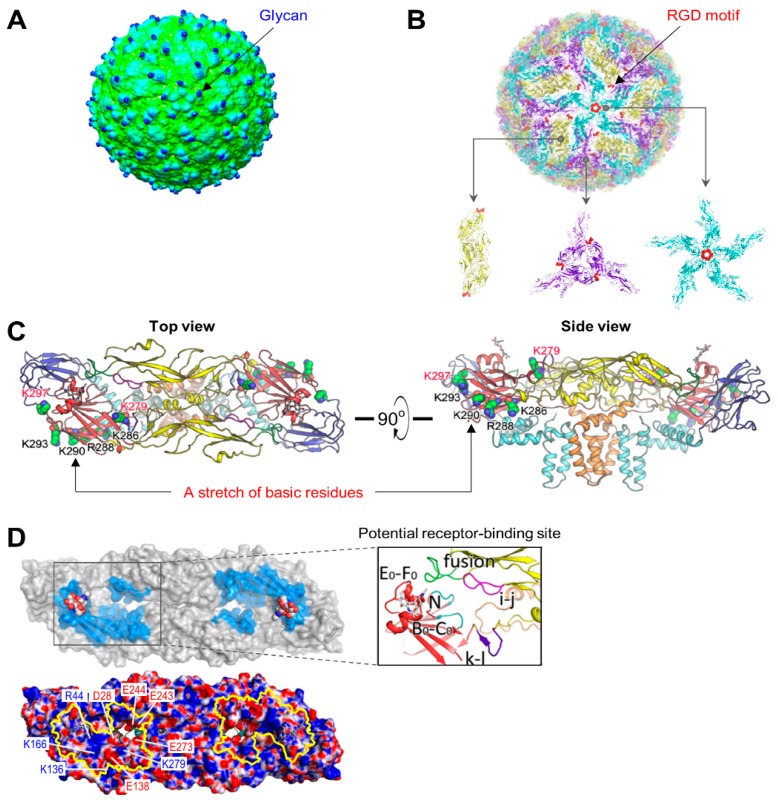
Location of the viral components involved in JEV entry on the protein shell of viral particles. (**A**) Location of glycans. The carbohydrate moieties attached to Asn^154^ are indicated on the surface of the cryo-EM map of JEV at a lower contour level. (**B**) Location of RGD motifs. RGD motifs are shown on the 2-, 3-, and 5-fold symmetry axes of the icosahedron. (**C**) Location of a basic residue-rich stretch in the E ectodomain. A cluster of six basic residues (Lys^279^, Lys^286^, Arg^288^, Lys^290^, Lys^293^, and Lys^297^) in the E ectodomain is shown on the top and side views of the atomic model of an E:M:M:E heterotetramer. The same color scheme as in Figure 3C is used, with the four resides (black) being buried inside the E ectodomain and the two resides (red) being exposed outside. (**D**) Location of putative receptor-binding sites. A pair of the charged residue-rich putative receptor-binding sites is highlighted in blue, with the glycans attached to Asn^154^ on the external surface of an E:M:M:E heterotetramer (top panel). The charged residues are indicated on the external electrostatic surface of the E:M:M:E heterotetramer, with the two putative receptor-binding sites outlined in yellow (bottom panel). An inset shows the molecular interactions formed in the putative receptor-binding site, involving the fusion and i-j loops that interact with the N-terminal loop of the A_0_ strand (labeled as N), the B_0_-C_0_ and E_0_-F_0_ loops in E-DI, and the k-l loop in E-DII from the opposite monomer. The images of this figure were graciously provided by Dr. Xiangxi Wang [176].

Despite recent advances in our understanding of the near-atomic resolution cryo-EM structure of JEV [176], the mechanisms by which the virion binds to its cellular receptors are not fully understood. In JEV [285,286,287] and other mosquito-borne flaviviruses [181,182,183,184,185,243,288,289,290,291,292,293,294], the presence of an N-linked glycan in E-DI (Figure 4A) and an RGD motif in E-DIII (Figure 4B) on the viral membrane suggests a mechanism of relatively nonspecific interactions with the carbohydrate-binding lectins and RGD-binding integrins on the cell surface, respectively; in agreement with this notion, blocking/alteration of either the N-glycosylation or RGD motif generally negatively affects viral entry to varying degrees, but fails to abolish the process [182,243,285,286,287,288,289,290,291,292,293,294,295]. Also, a string of six closely dispersed basic residues (Lys^279^ to Lys^297^) located in the last strand I_0_ of E-DI and the linker between E-DI and E-DIII (Figure 4C), conserved among the members of the JE and DEN serogroups, has been proposed as a potential binding site for glycosaminoglycans (GAGs) [180,295]. However, the cryo-EM structure of JEV indicates that, of the six basic residues, four central residues are buried, suggesting that conformational changes are required to make this potential GAG-binding site accessible to GAGs [176]. Most intriguingly, the cryo-EM structure of JEV, combined with a structure-based amino acid sequence alignment of the E proteins from seven different flaviviruses (JEV, WNV, MVEV, SLEV, ZIKV, DENV, and YFV), reveals an unusual “hole” on the viral surface, with distinct electrostatic characteristics (Figure 4D) that could be a potential receptor-binding site for JEV and other members of the JE serogroup [176,296]. Thus, the viral components and their interacting cellular counterparts required for triggering flavivirus internalization after binding on the cell surface are still elusive.

#### 4.2.2. Cellular Components

JEV maintains a natural transmission cycle among birds, pigs, and other vertebrate hosts, with mosquito vectors; in vitro, JEV can infect and replicate in a broad range of cell types originating from many different vertebrate and invertebrate species [165], suggesting that there is probably more than one host factor responsible for viral entry. The host factors documented to be involved in the early steps of JEV entry to date are summarized in Table 1.

##### Glycosaminoglycans (GAGs)

In JEV [297,298,299,300] as well as six other pathogenic flaviviruses [180,274,300,301,302,303,304,305,306,307,308,309,310,311,312], GAGs, a family of linear, polydisperse, sulfated polysaccharides [313] (such as the heparan sulfates found in all animal tissues), serve as one of the initial attachment factors for concentrating viral particles on the cell surface prior to the interaction with other molecules. Negatively charged sulfate groups on the GAGs can bind to a cluster of positively charged residues on the viral E glycoprotein [314]. In the case of JEV, a role for GAGs in viral attachment has been demonstrated by (i) competition for viral binding to hamster kidney-derived BHK-21 cells by highly sulfated GAGs, such as heparin and dextran sulfate; (ii) pretreatment of BHK-21 cells with sodium chlorate, a potent sulfation inhibitor; (iii) a comparison of the binding efficiency of the virus to the hamster ovary-derived wild-type CHO cell line and its mutants with defects in GAG biosynthesis; and (iv) the identification of single net-positive-charge amino acid changes (e.g., E^49^K, E^138^K, E^306^K, D^389^G/D^389^N, and E^390^G) in the E-DI or E-DIII region, with an enhanced binding capacity for GAGs [297,298,299,300,315]. Interestingly, most if not all cell culture-adapted JEVs, including a live-attenuated JE vaccine SA_14_-14-2 strain derived from its virulent parental SA_14_ strain, exhibit an increased ability to bind heparin, a highly sulfated GAG [274,300,315]. Similar results have also been observed for the live-attenuated YF vaccine 17D strain derived from its virulent parental Asibi strain [301]. Thus, an increased binding affinity for highly sulfated GAGs seems to be favorable for flavivirus growth in cell culture, and GAG-adapted flaviviruses tend to show attenuated phenotypes in vivo.

##### C-Type Lectins

One set of attachment factors involved in flavivirus entry is the family of C-type lectins, which are Ca^2+^-dependent glycan-binding proteins that recognize carbohydrate moieties on the surface of invading pathogens, act as the receptors for internalization, and deliver the pathogens to endosomes for antigen presentation, thereby activating host defense systems [316,317]. Of particular interest are (i) the dendritic cell-specific intercellular adhesion molecule (ICAM)-3-grabbing non-integrin (DC-SIGN, also called CD209 and CLEC4L [318,319]), which is highly expressed on subsets of dendritic cells (DCs) and macrophages; and (ii) the liver/lymph node-specific ICAM-3-grabbing non-integrin (L-SIGN, also known as CD209L, CLEC4M, and DC-SIGNR for “DC-SIGN-related” [320,321,322]), which is mainly expressed on endothelial cells in the liver and lymph nodes [323]. For JEV, siRNA knockdown and antibody blocking experiments, combined with the characterization of a DC-SIGN mutant defective in its internalization, have shown that DC-SIGN is important for viral binding to DCs that is mediated by an N-linked mannose-rich glycan at Asn^154^ on the viral E protein, but it is dispensable for subsequent internalization [287]. Similarly, for WNV [181,324] and DENV [182,184,325,326,327,328,329], both DC-SIGN and L-SIGN have been shown to promote infection via an interaction with an N-glycan(s) at Asn^154^ (for WNV) and at both Asn^67^ and Asn^153^ (for DENV) on the viral E protein.

Based on the work with WNV and DENV, the use of DC-SIGN and L-SIGN as attachment factors for flaviviruses varies, depending on the number and location of N-glycosylation sites on the viral E protein [182,183,324,325], as well as on the type of N-glycans linked to these sites, which is determined by the cells used for virus production [181,324,325,326]. Although glycosylation profiles may vary in a given cell line, in general, the high-mannose N-glycans on mosquito cell-produced virions are recognized well by both DC-SIGN and L-SIGN, whereas the complex N-glycans on mammalian cell-produced virions are preferentially recognized by L-SIGN. There is a further added level of complexity because “mosaic” partially mature flaviviruses contain a small, but detectable, amount of the glycosylated uncleaved prM proteins [330,331], with one to three potential N-glycosylation sites within the pr region [332] that may directly or indirectly contribute to the lectin-mediated attachment of flaviviruses to the cell surface [324,325,326]. Notably, the physiologically relevant functional importance of DC-SIGN in flavivirus replication and pathogenesis in humans is underlined by the association of a single nucleotide polymorphism (SNP) found in the promoter region of the DC-SIGN gene with a greater susceptibility to dengue hemorrhagic fever (SNP rs4804803 [333]) and severe forms of tick-borne encephalitis (SNP rs2287886 [334]) in certain subpopulations.

There are three other C-type lectins that have been suggested to participate in the early steps of JEV infection: mannose receptor (MR); C-type lectin domain family 5, member A (CLEC5A); and liver and lymph node sinusoidal endothelial cell C-type lectin (LSECtin) [286,335,336,337]. (1) MR is expressed on subsets of macrophages and DCs, as well as on nonvascular endothelium; it plays multiple important roles in clearing endogenous molecules, promoting antigen presentation, and modulating cellular activation and trafficking [338]. A study has shown that the extracellular region of MR binds broadly to mosquito cell-produced DENV particles and pointedly to mammalian cell-expressed DENV E ectodomains in a Ca^2+^-dependent manner; in addition, the cell surface expression of human MR in mouse embryo-derived 3T3 cells confers DENV binding, and anti-MR antibodies inhibit DENV infection in human macrophages [335]. The same study has also reported that the extracellular region of MR binds in enzyme-linked immunosorbent assays to formalin-inactivated JEV and TBEV, although the nature of this binding has not been characterized [335]. (2) CLEC5A is exclusively expressed on myeloid cells (e.g., macrophages and monocytes) and associates with a 12-kDa DNAX-activating protein (DAP12), an adaptor molecule that transduces intracellular signaling involved in innate immunity; thus, CLEC5A is also known as myeloid DAP12-associating lectin-1 (MDL-1) [339,340]. CLEC5A has been shown to interact directly with JEV [336] and DENV [337], albeit in a Ca^2+^-independent manner, and is capable of inducing DAP12 phosphorylation in macrophages. Unlike DC-SIGN and L-SIGN, the CLEC5A-JEV/DENV interaction does not promote viral infection, but rather stimulates the release of proinflammatory cytokines (e.g., TNF-α and MCP-1), thereby potentially contributing to the pathogenesis of virus-induced inflammatory diseases [336,337]. In the case of both JEV and DENV infection, the inflammation-associated viral pathogenesis and lethality in mice can be ameliorated by blocking the CLEC5A-JEV/DENV interaction with anti-CLEC5A antibodies [336,337]. (3) LSECtin (also known as CLEC4G) is expressed on myeloid cells, as well as on sinusoidal endothelial cells of the liver and lymph node; it mediates pathogen recognition, uptake, and internalization [317,341]. Using human B lymphocyte-derived Daudi cells that are non-susceptible to JEV infection, researchers have recently shown that the ectopic expression of LSECtin renders the cells susceptible to JEV infection. This infection can be inhibited by N-acetylglucosamine β1–2 mannose (a target for LSECtin) but not by mannan (a target for DC-SIGN/L-SIGN) [286]; however, the underlying mechanism of LSECtin in JEV entry has not been fully defined.

In addition to the mammalian C-type lectins described above, a family of mosquito galactose-specific C-type lectins (mosGCTLs) has been reported to play a central role in the entry steps of JEV [342], WNV [343], and DENV [344] in their major *Culex*/*Aedes* mosquito vectors. The original work has shown that mosGCTL-1 (VectorBase accession no. AAEL000563) as a secreted form of mannose-binding lectin (MBL) binds to WNV in a viral E protein-mediated Ca^2+^-dependent manner and brings the mosGCTL-1-WNV complex to its cell surface receptor, mosquito protein tyrosine phosphatase-1 (mosPTP-1), thereby facilitating WNV entry both in vivo and in vitro [343]. The mosPTP-1 is a mosquito homolog of human CD45 that is expressed on all nucleated cells of hemopoietic origin [345] and is critical for thymocyte development and activation [346,347] through its interaction with human MBL [348]. Since CD45 is expressed on hematopoietic cells that are important for flavivirus pathogenesis and host immunity [349], it will be interesting to determine whether the human MBL-CD45 interaction can also mediate the entry of flaviviruses into human cells [350]. Moreover, mosquito blood-feeding experiments have demonstrated that WNV infection can be blocked in vivo with anti-mosGCTL-1 antibodies [343], suggesting a promising new approach to interrupt the life cycle of WNV in mosquito populations. Similarly, mosGCTL-7 (AAEL002524) and mosGCTL-3 (AAEL000535) have subsequently been shown to be able to mediate the mosquito cell entry of JEV and DENV, respectively [342,344]. For DENV infection, a genetic association of the exon 1 polymorphisms of the human MBL gene (MBL2) with dengue hemorrhagic fever has been suggested because variant alleles and haplotypes related to low production levels of MBL are associated with the severity of DENV-induced diseases [351]. Further investigation is required to elucidate the underlying mechanism behind the variation in the usage of specific mosGCTLs for particular flaviviruses, along with distinct mosPTPs, and the role of the mosGCTL-mosPTP pathway in flavivirus entry.

##### Integrins

Integrins are a family of cell surface receptors, each composed of two subunits (α and β), that act as linkers between the extracellular matrix and the actin cytoskeleton, and play a critical role in the activation and homing of hematopoietic cells [352]. Biochemical and molecular studies have demonstrated that in monkey kidney-derived Vero or human cervical carcinoma HeLa cells, the lineage-2 Sarafend strain of WNV binds to α_v_β_3_ integrin, and WNV infection is notably decreased by pretreatment with anti-α_v_β_3_ antibodies, competition with recombinant α_v_ or β_3_ protein, or siRNA knockdown of the β_3_ subunit; however, somewhat unexpectedly, WNV infection is only marginally affected by pretreatment with synthetic RGD peptides, an inhibitor of integrin-ligand interactions [288,353]. It has also been noted that soluble α_v_β_3_ can block WNV infection of Vero cells in a dose-dependent manner, and the expression of α_v_β_3_ increases the susceptibility to WNV infection of hamster melanoma CS-1 cells lacking functional integrin [288]. In contrast, another study has shown that the lineage-1 NY385-99 strain of WNV can infect and replicate in mouse embryonic fibroblasts lacking functional α_v_β_3_ [354]. Therefore, the discrepancies in these studies suggest that the role of α_v_β_3_ in WNV entry is potentially strain-specific and/or cell type-dependent. Further investigation is needed to define a potential role of α_v_β_3_ in WNV infection [355]. As previously seen for WNV, pretreatment with anti-α_v_β_3_ antibodies has been shown to inhibit JEV entry into Vero cells [288]. A potential role for α_v_β_3_ in JEV entry has also been proposed in hamster kidney-derived BHK-21 cells, based on shRNA-based gene silencing and antibody/peptide-based blocking experiments using anti-α_v_β_3_ antibodies and synthetic RGD peptides, although their inhibitory effects varied significantly [285].

##### Other Host Factors

Heat shock proteins (HSPs) were long believed to be cytoplasmic proteins, but their protein- and/or lipid-mediated association with intracellular and plasma membranes is now well documented [356]. To date, two families of HSPs have been proposed to participate in the early steps of JEV infection, possibly in a cell type-dependent manner: (i) three members of the HSP70 family, namely the prototype HSP70, heat shock cognate protein 70 (HSC70), and glucose-regulated protein 78 (GRP78, also referred to as BiP for “binding immunoglobulin protein”); and (ii) the prototype HSP90 of the HSP90 family [196,357,358,359,360,361,362]. The details are as follows: (1) HSC70 derived from *Ae. albopictus* C6/36 cells has been shown by co-immunoprecipitation experiments to bind to JEV [357]. In C6/36 cells, gene knockdown experiments have identified HSC70 isoform D, which is involved in the clathrin-mediated endocytosis of JEV [196]. (*2*) HSP70 derived from mouse neuronal Neuro-2a cells has been shown by virus overlay protein binding assays to interact with JEV; the interaction between HSP70 and the JEV E protein has been demonstrated by co-immunoprecipitation and immunoblotting [358]. Antibody blocking experiments using anti-HSP70 antibodies have produced a significant reduction in JEV entry into Neuro-2a cells [358]. (3) In human hepatoma Huh7 cells, the association of both HSP70 and JEV E proteins with cholesterol-rich lipid rafts on the cell surface has been shown to be critical for JEV infection [197,359]. In Huh7 cells, both antibody blocking and siRNA knockdown experiments have revealed that HSP70, but not HSC70 or GRP78, is crucial for the host cell entry of vesicular stomatitis virus-based pseudoviruses expressing JEV prM and E proteins [359]. (4) In Neuro-2a cells, however, a combination of biochemical, genetic, and molecular experiments has shown that GRP78, capable of interacting with JEV E-DIII, plays multiple roles in the entry and post-entry steps of JEV infection [360]. (5) HSP90 isoform HSP90β, but not HSP90α, is co-localized and co-immunoprecipitated with JEV E proteins in hamster kidney-derived BHK-21 cells infected with JEV; HSP90β is also shown to be secreted into the culture supernatant from JEV-infected BHK-21 cells, presumably in association with released virus particles, promoting viral infectivity or the release of infectious particles [361]. Thus, all the data available to date suggest that several members of the HSP70 and HSP90 families have pivotal, isoform-specific, and differential roles in JEV entry, depending to some extent on the cell type, like those documented in DENV entry [363,364,365,366,367,368,369,370].

Several other host factors are thought to promote the infection of various cell types by JEV: (i) the 37/67-kDa high-affinity laminin receptor, CD4, and CD14 in mouse microglial BV-2 cells [362]; (ii) the type III intermediate filament vimentin in mouse neuroblastoma N18 and human neuroblastoma HTB-11 cells [371,372], as well as in porcine kidney PS cells [373]; (iii) the low-density lipoprotein receptor in hamster kidney-derived BHK-21 cells [374]; (iv) a 74-kDa protein in monkey kidney-derived Vero cells [375]; and (v) a 53-kDa protein in *Ae. albopictus* C6/36 cells [376]. However, the biological function and physiological role of these molecules in JEV entry remain to be defined. In addition, a handful of other cellular components have also been put forward as putative receptors for one or more of other mosquito-borne flaviviruses, such as WNV, ZIKV, DENV, and YFV: (a) the phosphatidylserine-recognizing TIM (TIM-1, -3, and -4) and TAM (TYRO3, AXL, and MER) family members [185,377,378,379,380,381,382,383,384,385,386,387,388], (b) the phosphatidylserine- and phosphatidylethanolamine-binding protein CD300a [389], (c) the tight junction component Claudin-1 [390,391], (d) the scavenger receptor class B type I coupled with apolipoprotein A-I [392], (e) the 37/67-kDa high-affinity laminin receptor [393,394], (f) CD14-associated molecules [395], (g) the carbohydrate β-*N*-acetylglucosamine moiety of glycosphingolipids [396,397], (h) the natural killer cell-activating receptor, NKp44 [398], and (i) the mosquito cell-derived prohibitin [399] (see two recent review articles for a detailed description of these molecules [186,400]). Despite these research efforts discussed above, however, the cell surface receptors and other host factors required for directing JEV, or any other flavivirus, into the receptor-mediated endocytic pathway and low pH-dependent membrane fusion are still unknown.

### 4.3. Endocytosis

JEV is internalized from the plasma membrane of host cells to an endosomal compartment via multiple endocytic routes in vitro [401], largely depending on the types of cell being infected: (i) the classical clathrin-dependent pathway observed in the mosquito-derived C6/36 [196], hamster kidney-derived BHK-21 [194], monkey kidney-derived Vero [198,201], porcine kidney-derived PK15 [195], and mouse neural stem-like C17.2 [197] cells; and (ii) the non-classical clathrin-independent pathway (e.g., caveolin-dependent pathway) observed in the human neuroblastoma SK-N-SH [202], mouse neuroblastoma Neuro-2a [201], and rat neuroblastoma B104 [203] cells. In almost all of these cell lines (BHK-21, Vero, PK15, C17.2, SK-N-SH, Neuro-2a, and B104), the depletion of cholesterol from the cell membrane with methyl-β-cyclodextrin reduces productive JEV infection [194,195,197,201,202,203], suggesting an important role for cholesterol and possibly cholesterol-rich lipid rafts in both clathrin-dependent and clathrin-independent endocytosis of JEV. Upon internalization, trafficking of the endocytosed vesicles containing JEV particles to early and recycling endosomes is demonstrated to be regulated by the two Rab GTPases, Rab5 and Rab11, for clathrin-dependent endocytosis in BHK-21 cells [194]. In the case of clathrin-independent endocytosis in Neuro-2a cells, JEV-carrying vesicles are transported to Rab5-positive early endosomes before the release of its genomic RNA into the cytosol, and this vesicle trafficking is shown to be mediated by the actin-myosin II machinery that is modulated by the major Rho GTPase RhoA [201]. Similarly, RhoA and Rac1 GTPase-mediated actin rearrangements are documented to be critical for caveolin-dependent endocytosis of JEV in SK-N-SH cells [202]. Considering the variations mentioned above, it is necessary to determine the main endocytic pathway co-opted by JEV for its entry into human neurons in the brain, which are the major target cells of JEV, and human monocytes and macrophages/DCs in the periphery, which are likely to be of importance in mediating neuroinvasion.

Among other flaviviruses, WNV is reported thus far to adopt the clathrin-mediated endocytic pathway for entry into C6/36 [190], Vero [191], and HeLa [402] cells. On the other hand, DENV is shown to be able to enter the cytosol via an endosomal compartment, not only predominantly by clathrin-dependent endocytosis (as described in C6/36 [189,192,403], Vero [205,206,404], BSC-1 [193], NIH3T3 [328], HeLa [193,402], A549 [404], Huh7 [199], HepG2 [200,405], and ECV304 [406] cells and human monocytes/immature DCs [328,407]) but also partially by clathrin-independent endocytosis (as described in Vero [204,205,206,404] and HepG2 [200] cells). It has been noted in Vero cells, however, that DENV can utilize both clathrin-dependent and clathrin-independent pathways for internalization, depending on the viral serotype, and the virus can be transported to a different endosomal compartment prior to membrane fusion, depending on the viral strain, even within the same serotype [205,404]. Moreover, the main endocytic route employed by DENV in Vero cells is reported to be altered from a clathrin-independent pathway for C6/36-grown virus to the clathrin-dependent pathway for Vero-adapted virus [206]. In the case of both DENV and WNV, numerous studies have indicated the functional importance of cholesterol [204,354,408], cytoskeleton and motor proteins [189,190,191,192,199,328,409,410], and Rab GTPase-regulated vesicle trafficking [193,199,204,205,328,402] in the process of their entry into various cell lines.

As is true for DENV, YFV is also able to enter HeLa cells via two distinct endocytic routes, as demonstrated by the finding that the wild-type virulent Asibi strain primarily utilizes the clathrin-dependent pathway, whereas its attenuated vaccine 17D strain exploits a pathway independent of both clathrin and caveolin [411]. A mutagenetic analysis has indicated that the strain-specific use of distinct endocytic pathways for YFV internalization is due to the 12 amino acid differences found within the viral E protein between Asibi and 17D [411]. Interestingly, the 17D vaccine strain is shown to enter HeLa and several other human cells more efficiently than does the parental Asibi strain, resulting in a stronger induction of the cytokine-mediated antiviral response [411]. These data suggest a potential link between viral entry and the host immune response. It will be interesting to examine whether the strain-specific use of different endocytic pathways for YFV internalization is maintained by an isogenic pair of JEV SA_14_ and SA_14_-14-2 strains. In summary, JEV and other flaviviruses enter a wide range of different host cells by viral E protein-directed endocytosis, but the precise endocytic pathway used for viral internalization is determined by a combination of both the genetic composition of the viral E protein and the availability of its interacting cellular components in a given cell type.

In cell biology, clathrin-mediated endocytosis is one of the best-studied processes, with a network of various cellular proteins well characterized to date [412]. These previously known host factors are generally required for those viruses that usurp the clathrin-mediated endocytic pathway [381,413,414,415,416,417,418]. In addition, the interferon-inducible glycosylphosphatidylinositol-anchored lymphocyte antigen 6E (LY6E) has been found to play a role in facilitating YFV and WNV infection [415,419]. A recent study has shown that LY6E is required for the clathrin-mediated uptake of several mosquito-borne flaviviruses (WNV, ZIKV, and DENV) and of transferrin-coated particles that are similar in size to these virions, but not of free transferrins [420]. Cell biological studies have revealed that the internalization of these virions and transferrin-coated particles is triggered by the formation of tubule-like structures of LY6E, which depend on the vacuolar ATPase-associated transmembrane protein RNASEK (for “ribonuclease kappa”) and microtubules [420,421,422]. It will be interesting to test whether this LY6E-mediated size-dependent endocytic pathway is also involved in the clathrin-mediated uptake of JEV. Furthermore, a genome-wide siRNA screen has identified the human G protein-coupled receptor kinase 2 (GRK2, also known as ADRBK1) as being involved in promoting both the entry and RNA replication steps of YFV and DENV [417]. Similar genomic screens using RNAi and CRISPR/Cas9 approaches have revealed a collection of human host factors that function at the early stages of DENV and ZIKV infection, of which the ER membrane protein complex (EMC) is suggested to play a role in a post-attachment step during viral entry [381]. Another large-scale RNAi screen has discovered a set of insect host factors and their human homologs that are required for DENV replication, with a subset presumably involved in viral entry [416]. Additional research is warranted to define the host factors selectively involved in the internalization process of JEV and other flaviviruses.

### 4.4. Membrane Fusion and Uncoating

**Figure 5:**
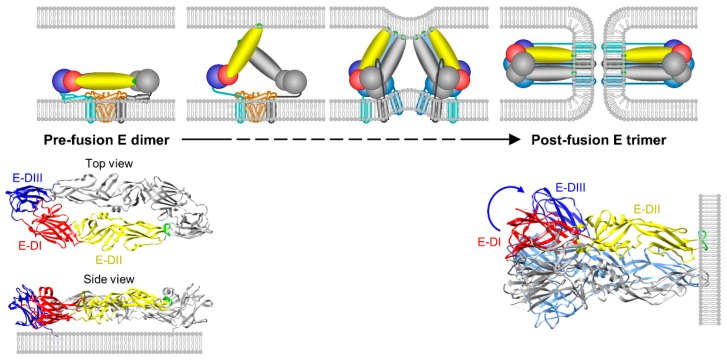
Schematic presentation illustrating JEV E-mediated membrane fusion. The membrane fusion event is initiated by the binding of a cellular receptor(s) to the viral pre-fusion E dimer of E:M:M:E heterotetramers, followed by dissociation of the E dimers, insertion of the fusion loops into the host cell membrane, trimerization of membrane-interacting E monomers, and changes in the E trimers to the post-fusion conformation. The diagram is modified from previously published work [442], with minor modification. Shown below the diagram are the pre-fusion E dimer of JEV (PDB 3P54) and the post-fusion E trimer of St. Louis encephalitis virus (SLEV, PDB 4FG0), a member of the JE serogroup.

As is true for other flaviviruses, JEV enters its host cells through clathrin-(in)dependent endocytosis and low pH-triggered membrane fusion, both of which are mediated by the viral glycoprotein E, a prototypical class II fusion protein [187,330,423,424,425]. In recent years, a working model of flavivirus membrane fusion has been established based on the dimeric pre-fusion and trimeric post-fusion E ectodomain crystal structures and biochemical properties of JEV [296,426], WNV [427,428], SLEV [429], ZIKV [430], DENV [210,211,240,431,432,433], and TBEV [208,278], together with reconstitution of in vitro transient fusion intermediates at different stages of the fusion process [207,209,212,213,214,434]. As illustrated in Figure 5, the fusion is initiated by a low pH-induced dissociation of the antiparallel E:M:M:E heterotetramers, followed by the exposure of the hydrophobic fusion loop of each E protein, its insertion only partway into the outer bilayer leaflet of the host cell membrane, and a large-scale structural rearrangement of the antiparallel E:E homodimer of the E:M:M:E heterotetramers into a parallel E:E:E homotrimer [330,423,425,435]; however, the fate of the antiparallel M:M homodimers is unknown. In the post-fusion E:E:E homotrimer, E-DIII folds back against the central trimer, presumably with the helical stem extended from the C-terminus of E-DIII along E-DII and toward the fusion loop (known as “zipping”) [264,265,436,437,438,439,440,441]. During this E-DIII fold-back process, the overall structures of E-DI, E-DII, and E-DIII are maintained, but the relative orientations of the three domains are rearranged. To date, the steps involved in the pH-induced domain rearrangement and their underlying mechanisms are not completely understood.

It is conceivable that cellular proteins may assist in bringing to completion the low pH-initiated membrane fusion and discharge of the viral nucleocapsid/genome into the cytoplasm, but they have not yet been explored extensively. Earlier, a human genome-wide siRNA screen identified a collection of host proteins involved in the early steps of WNV infection; one of these proteins, ubiquitin ligase CBLL1 (Cbl-like protein 1, also known as Hakai), in conjunction with the ubiquitin-proteasome system (UPS), has been suggested to play a critical role in a post-attachment step of WNV entry, such as internalization [415]. The UPS has also been implicated in JEV entry, especially in a post-attachment step prior to the initial translation of viral genomic RNA [443]. Interestingly, biochemical assays, combined with live-cell imaging and single-particle tracking, have shown that in some flaviviruses (JEV and YFV), the virus-host cell membrane fusion is a distinct event that precedes the microtubule-mediated release of viral nucleocapsid/genome into the cytoplasm [444]. Even more intriguingly, a recent molecular biology-based study has demonstrated that during DENV entry, the release of viral genome or uncoating of viral nucleocapsid is hampered by inhibiting ubiquitination [445]. In the same study, viral capsid was shown to be degraded after internalization by the host UPS, although this event was not required to release the viral genomic RNA into the cytoplasm for initial translation [445]. On the other hand, another study has reported that the siRNA-based knockdown of CBLL1 expression has no significant effect on the infection efficiency of several mosquito-borne flaviviruses (WNV, DENV, and YFV) in human cells, and that treatment with proteasome inhibitors (MG132 and lactacystin) has no measurable impact on the entry of WNV [446]. Thus, further investigation is needed to determine the precise role of CBLL1 and UPS in the host-cell entry of JEV and other flaviviruses.

## 5. Conclusions: JEV Entry Is an Area of High Interest for Future Research

Viral entry is the first step in an infection process that involves a cascade of multiple, highly coordinated interactions between the virus and its target cells. This aspect of virus research is of particular relevance because viral entry is a common feature essential to the initiation, dissemination, and maintenance of productive infection by all human and animal viruses. Whereas the viral factors involved in JEV entry are well defined (particularly viral glycoprotein E, which is involved in attachment, endocytosis, and membrane fusion), the host factors that participate in this multistep process remain poorly understood. To date, three types of multiple host factors (GAGs, C-type lectins, and integrins), along with their interacting counterparts (basic residue-rich region, glycan, and RGD motif, respectively) in the viral E glycoprotein, have been relatively well characterized as attachment factors for a range of mammalian and/or mosquito cells for promoting the cell entry of JEV and other flaviviruses, although they often act in a strain-specific and cell type-dependent manner. However, the host entry factor(s) that can direct receptor-mediated endocytosis and low pH-dependent membrane fusion once viral attachment has occurred remain elusive. In particular, identification of the bona fide cell-surface receptor(s) responsible for JEV internalization has been a major challenge in JEV biology, primarily because of a lack of availability of the nonsusceptible cell line that has a block in JEV entry but can fully support the subsequent post-entry steps (translation, RNA replication, assembly, and release) and is therefore capable of serving as a platform cell line for receptor screening and validation. Over the years, only a few cell lines have been described to be resistant to JEV infection (e.g., human B lymphoblast Daudi [286] and mouse neuroblastoma N18TG2 [447]). With the use of such a JEV-nonsusceptible cell line, two complementary genome-wide genetic screens for both the gain- and loss-of-function of JEV entry can allow us to identify the host factors that are critical for JEV entry and to dissect the discrete entry steps that are regulated by specific host factors. The outcomes of this research will not only shed new light on the cell/tissue tropism and pathogenesis of JEV, and possibly other closely related encephalitic flaviviruses, but also provide new targets for the development of novel antiviral interventions capable of inhibiting the early steps of JEV infection.

## Figures and Tables

**Table 1:** Cellular components involved in the early steps of JEV entry.

Cellular Component	Type/Property	Host Cell	Virus
Heparan sulfate	Glycosaminoglycan	Mammal	JEV, WNV, MVEV, ZIKV, DENV, YFV, TBEV
DC-SIGN/L-SIGN	C-type lectin	Mammal	JEV, WNV, DENV
MR	C-type lectin	Mammal	JEV, DENV, TBEV
CLEC5A	C-type lectin	Mammal	JEV, DENV
LSECtin	C-type lectin	Mammal	JEV
mosGCTL-7, 1, and 3	C-type lectin	Mosquito	JEV, WNV, DENV
α_v_β_3_	Integrin	Mammal	JEV, WNV
HSP70/HSC70/GRP78	70-kDa heat shock protein	Mammal, Mosquito	JEV, DENV
HSP90	90-kDa heat shock protein	Mammal	JEV, DENV
37/67-kDa LR	High-affinity laminin receptor	Mammal	JEV
CD4	Immunoglobulin superfamily	Mammal	JEV
CD14	Pattern recognition receptor	Mammal	JEV
Vimentin	Type III intermediate filament	Mammal	JEV
LDLR	Low-density lipoprotein receptor	Mammal	JEV
74-kDa protein	Not characterized	Mammal	JEV
53-kDa protein	Not characterized	Mosquito	JEV
